# A framework for assessing the consistency of drug classes across sources

**DOI:** 10.1186/2041-1480-5-30

**Published:** 2014-07-09

**Authors:** Rainer Winnenburg, Olivier Bodenreider

**Affiliations:** 1 Lister Hill National Center for Biomedical Communications, National Library of Medicine, Bethesda, MD, USA

**Keywords:** Drug classes, MeSH, ATC, Instance-based mapping, Lexical mapping

## Abstract

**Background:**

The objective of this study is to develop a framework for assessing the consistency of drug classes across sources, such as MeSH and ATC. Our framework integrates and contrasts lexical and instance-based ontology alignment techniques. Moreover, we propose metrics for assessing not only equivalence relations, but also inclusion relations among drug classes.

**Results:**

We identified 226 equivalence relations between MeSH and ATC classes through the lexical alignment, and 223 through the instance-based alignment, with limited overlap between the two (36). We also identified 6,257 inclusion relations. Discrepancies between lexical and instance-based alignments are illustrated and discussed.

**Conclusions:**

Our work is the first attempt to align drug classes with sophisticated instance-based techniques, while also distinguishing between equivalence and inclusion relations. Additionally, it is the first application of aligning drug classes in ATC and MeSH. By providing a detailed account of similarities and differences between drug classes across sources, our framework has the prospect of effectively supporting the creation of a mapping of drug classes between ATC and MeSH by domain experts.

## Background

### Motivation and objectives

Drug classes provide a convenient mechanism for organizing drugs in terms of chemical structure (e.g., *Sulfonamides–*a group of compounds that contain the structure SO_2_NH_2_), function (e.g., *Anti-Bacterial Agents–*often referred to as antibiotics), mechanism of action (e.g., *Hydroxymethylglutaryl-CoA Reductase Inhibitors–*a group of drugs, also called statins, which block an enzyme involved in the production of cholesterol in the liver), metabolism (e.g., *inhibitors of CYP2C9–*drugs that block an enzyme from the Cytochrome P450 protein family, which is involved in the metabolism of drugs, such as *ibuprofen* and *fluoxetine*, and whose activity is influenced by other drugs, such as *rifampicin* and *fluconazole*), and adverse events (e.g., *drugs that induce QT prolongation–*the antimalarial drug *halofantrine* slows down ventricular repolarization, which predisposes to certain types of arrhythmias). The interested reader is referred to [1] for more details about drug classes.

Several drug classifications have been developed for different purposes. For example, the Anatomical Therapeutic Chemical (ATC) classification of drugs supports pharmacoepidemiology, while the Medical Subject Headings (MeSH) is oriented towards the indexing and retrieval of the biomedical literature [2,3]. Moreover, sources tend to provide different lists of drug classes, and such lists tend to be organized in different ways according to the purpose of a given source. For example, the ATC uses a complex classificatory principle, in which the first subdivision is primarily anatomical (i.e., distinction based on the target organs or anatomical systems–e.g., *cardiovascular system drugs* vs. *dermatologicals*), followed by a therapeutic subdivision (i.e., therapeutic intent of the drugs in each anatomical group–e.g., *antibacterial drugs* vs. *antiviral drugs*), followed by a chemical subdivision (i.e., distinction between the structural and functional characteristics of drugs within a therapeutic subgroup–e.g., *macrolides*, such as *erythromycin*, vs. *fluoroquinolones*, such as *ciprofloxacin*, among the *antibacterial drugs*). On the other hand, MeSH maintains two parallel classifications, one based on chemical structure (e.g., *ciprofloxacin* is represented under *fluoroquinolones*), and one based on functional characteristics, including mechanism of action, physiologic effect and therapeutic use. (e.g., *ciprofloxacin* is linked to the mechanism of action *Topoisomerase II Inhibitors* and to the therapeutic use *Anti-Bacterial Agents*). In contrast to ATC, MeSH does not make distinctions based on the target anatomical location of the drug (e.g., there are two *Fluoroquinolones* classes for ophthalmological use vs. for systemic use in ATC, but only one *Fluoroquinolones* class in MeSH).

Ideally, drug classes with similar names should have similar members and drug classes with similar members should have similar names. In practice, however, the same name can be used to refer to different classes. For example, in ATC, *Fluoroquinolones* refers to both a set of ophthalmological drugs (8 members) and a set of systemic drugs (20 members), while, in MeSH, it refers to over 50 chemical compounds with similar structural properties. In the absence of an authoritative reference for drug classes, the task of determining when two classes are equivalent across sources remains extremely challenging. At the same time, the use of multiple classifications is often required in applications. This is increasingly the case as the use of ATC for pharmacovigilance is on the rise (e.g., [4]).

The objective of this study is to develop a framework for assessing the consistency of drug classes across sources, leveraging multiple ontology alignment techniques. This framework is meant to assist experts in the curation of a mapping between drug classes across sources. We present two applications of this framework, one to the alignment of drug classes between MeSH and ATC, and the other to the integration of MeSH and ATC drug class hierarchies. To our knowledge, this work represents the first effort to align drug classes between MeSH and ATC using a sophisticated instance-based alignment technique. Moreover, we propose metrics for assessing not only equivalence relations between classes, but also inclusion relations.

### Application of ontology alignment techniques to drug classes

The broad context of this study is that of ontology alignment (or ontology matching). Various techniques have been proposed for aligning concepts across ontologies, including lexical techniques (based on the similarity of concept names), structural techniques (based on the similarity of hierarchical relations), semantic techniques (based on semantic similarity between concepts), and instance-based techniques (based on the similarity of the set of instances of two concepts). An overview of ontology alignment is provided in [5]. The main contribution of this paper is not to propose a novel technique, but rather to apply existing techniques to a novel objective, namely aligning drug classes between MeSH and ATC. To this end, we use lexical and instance-based techniques, because the names of drug classes and the list of drugs that are members of these classes are the main two features available in these resources.

#### Lexical techniques

Lexical techniques compare concept names across ontologies and are a component of most ontology alignment systems [5]. When synonyms are available, they can be used to identify additional matches. Matching techniques beyond exact match utilize edit distance or normalization to account for minor differences between concept names.

As part of the Unified Medical Language System (UMLS), linguistically-motivated normalization techniques have been developed specifically for biomedical terms [6]. UMLS normalization abstracts away from inessential differences, such as inflection, case and hyphen variation, as well as word order variation. The UMLS normalization techniques form the basis for integrating terms into the UMLS Metathesaurus, but can be applied to terms that are not in the UMLS. For example, the ATC class *Thiouracils* (*H03BA*) and the MeSH class *Thiouracil* (*D013889*) match after normalization (ignoring singular/plural differences).

Lexical techniques typically compare the names of concepts across two ontologies as provided by these ontologies. However, additional synonyms can be used, for example, synonyms from the UMLS Metathesaurus. In other words, we leverage cosynonymy similarity for matching drug classes. In this case, although the ATC class *Anticholinesterases* (*N06DA*) and the MeSH class *Cholinesterase Inhibitors* (*D002800*) do not match lexically, both names are cosynonyms, because they are found among the synonyms of the UMLS Metathesaurus concept *C0008425*.

While there have been attempts to map individual drugs from ATC to concepts in the UMLS and MeSH through lexical techniques, [7] note that these techniques are not appropriate for the mapping of drug classes.

#### Instance-based techniques

Also called extensional techniques, instance-based techniques compare classes based on the sets of individuals (i.e., instances) of each class. While instance-based techniques are also available in many ontology alignment systems, the applicability of this technique is limited, because most biomedical ontologies consist of class hierarchies, but do not contain information about instances. Here, however, individual drugs (e.g., *atorvastatin*) are the members–not subclasses–of drug classes (e.g., *statins*). In other words, drug classes have individual drugs as instances, not subclasses and are therefore amenable to instance-based alignment techniques.

Several methods have been proposed to implement instance-based matching. [8] decompose these methods into three basic elements: (1) A measure is used for evaluating the association between two classes based on the proportion of shared instances. Typical measures include information-based measures (e.g., Jaccard similarity coefficient) and statistical measures (e.g., log likelihood ratio). (2) A threshold is applied to the measures and pairs of classes for which the measure is above the threshold are deemed closely associated and mapping candidates. (3) Hierarchical relations in the two ontologies to be aligned can also be leveraged by deriving instance-class relations between instances of a given class and the ancestors of this class. In other words, in addition to asserted classes (i.e., the classes of which individual drugs are direct members), we also consider inferred classes (i.e., the classes of which asserted classes are subclasses). For example, the class asserted in MeSH for the drug *atorvastatin* is *Hydroxymethylglutaryl-CoA Reductase Inhibitors* (i.e., *statins*), whose parent concepts include *Anticholesteremic Agents*. Therefore, the class *Anticholesteremic Agents* is an inferred drug class for *atorvastatin*.

To our knowledge, our work is the first attempt to align drug classes with instance-based techniques (i.e., beyond name matching), and the first application of aligning drug classes in ATC and MeSH. Moreover, while most ontology alignment systems mainly consider matches between equivalent classes, we are also interested in identifying those cases where one class is included in another class.

### Related work on drug classes, MeSH and ATC

In previous work, we compared drug classes between the National Drug File-Reference terminology (NDF-RT) and SNOMED CT from the perspective of semantic mining [9]. We also used an instance-based alignment technique, but only considered overlap between classes, not inclusion. Lexical alignment of the classes was not performed. Overall, we found that the overlap between NDF-RT and SNOMED CT classes was very limited. In [10], we mapped selected drug classes between NDF-RT and ATC through lexical techniques, observed the limitations of lexical techniques for the alignment of drug classes (also noted by [7]), and argued that the alignment could be improved by identifying mappings between the drugs in these classes.

As part of the EU-ADR project, [11] extracted adverse drug reactions from the biomedical literature and mapped MeSH drugs to ATC through the UMLS. However, their mapping was limited to individual drugs and did not include drug classes. The alignment of drug classes is one element of the broader integration of drug information sources in systems, such as the one developed by [12]. However, the preliminary version of their system integrates ATC, NDF-RT, RxNorm and the Structured Product labels, but not MeSH or the biomedical literature.

### Resources

Our framework leverages several knowledge sources. In addition to ATC and MeSH, the two sources of drug classes we propose to align and from which we extract information about drug-class membership, we also take advantage of RxNorm for aligning and normalizing individual drugs, and of the UMLS Metathesaurus as a source of synonymy for the lexical mapping of drug class names.

#### Anatomical Therapeutic Chemical Drug Classification System (ATC)

The ATC is a clinical drug classification system developed and maintained by the World Health Organization (WHO) as a resource for drug utilization research to improve quality of drug use [2]. The system is organized as a hierarchy that classifies clinical drug entities at five different levels: 1st level anatomical (e.g., *C*: *Cardiovascular system*), 2nd level therapeutic (e.g., *C10*: *Lipid modifying agents*), 3rd level pharmacological (e.g., *C10A*: *Lipid modifying agents, plain*), 4th level chemical (e.g., *C10AA*: *HMG CoA reductase inhibitors*), and 5th level chemical substance or ingredient (e.g., *C10AA05*: *atorvastatin*). The 2013 version of ATC integrates 4,516 5th-level drugs and 1,255 drug groups (levels 1-4). We refer to these drug groups as “ATC classes”.

#### Medical Subject Headings (MeSH)

The Medical Subject Headings (MeSH) is a controlled vocabulary produced and maintained by the NLM [3]. It is used for indexing, cataloging, and searching the biomedical literature in the MEDLINE/PubMed database, and other documents. The MeSH thesaurus includes 26,853 descriptors (or “main headings”) organized in 16 hierarchies (e.g., *Chemical and Drugs*). Additionally, MeSH provides about 210,000 supplementary concept records (SCRs), of which many represent chemicals and drugs (e.g., *atorvastatin*). Each SCR is linked to at least one descriptor through a “heading mapped to” relation (e.g., *atorvastatin* is associated with *Heptanoic Acids* and *Pyrroles*). These descriptors “mapped to” generally denote the chemical structure of the drug. While most chemical descriptors provide a structural perspective on drugs, some descriptors play a special role as they can be used to annotate the functional characteristics of drug descriptors and SCRs through a *pharmacologic action* relation (e.g., *atorvastatin* is linked to the mechanism of action *Hydroxymethylglutaryl-CoA Reductase Inhibitors* and to the therapeutic use *Anticholesteremic Agents*). MeSH 2013 is used in this study.

#### RxNorm

RxNorm is a standardized nomenclature for medications produced and maintained by the U.S. National Library of Medicine (NLM) [13]. RxNorm concepts are linked by NLM to multiple drug identifiers for commercially available drug databases and standard terminologies, including MeSH and ATC. (While RxNorm integrates drugs and classes from ATC and drugs from MeSH, it does not integrate classes from MeSH.) RxNorm serves as a reference terminology for drugs in the U.S. The August 2013 version of RxNorm used in this study integrates 10,108 substances, including ingredients (IN) and precise ingredients (PIN). Ingredients generally represent base forms (e.g., *atorvastatin*), while precise ingredients tend to represent esters and salts (e.g., *atorvastatin calcium*). RxNorm also represents clinical drugs, i.e., the drugs relevant to clinical medicine (e.g., *atorvastatin 10 MG Oral Tablet*). The relations among the various drug entities are represented explicitly in RxNorm (e.g., between ingredients and precise ingredients, and between ingredients and clinical drugs). NLM also provides an application programming interface (API) for accessing RxNorm data programmatically [14].

### Unified Medical Language System (UMLS)

The UMLS is a terminology integration system created and maintained by the National Library of Medicine (NLM) [15]. The UMLS Metathesaurus integrates over 150 terminologies, including MeSH, but not ATC. Synonymous terms across terminologies are grouped into concepts and assigned the same concept unique identifier. The Metathesaurus provides a comprehensive set of synonyms for biomedical concepts, including drug classes, and is often used for integrating terminologies beyond its own (e.g., [16]). Therefore, the UMLS is a useful resource for mapping class names from ATC to drug class concepts present in the source vocabularies of the Metathesaurus. NLM provides an application programming interface (API) for accessing UMLS data programmatically. Version 2013AA of the UMLS is used in this study^a^.

## Methods

**Figure 1:**
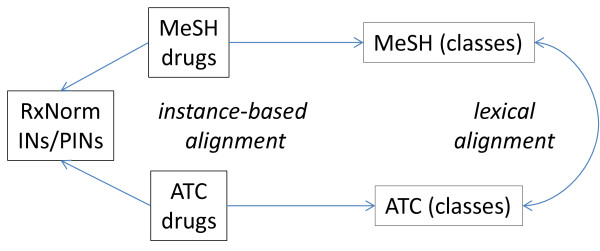
Alignment of ATC and MeSH classes.

Our framework for assessing the consistency of drug classes across sources (here MeSH and ATC) uses techniques for aligning drug classes based on their names and drug instances as depicted in Figure 1. It can be summarized as follows. Having established a reference list of drugs and drug classes, we compare the drug classes between MeSH and ATC based on their names (lexical alignment, Figure 1, right) and on the individual drugs these classes contain as members (instance-based alignment, Figure 1, left). Toward this end, we leverage similarity measures to compare the set of drugs in a class to the set of drugs in another class from the dual perspective of equivalence and inclusion. Finally, we compare the alignments obtained by the two approaches.

### Establishing a common reference for drugs, drug classes and drug-class members

#### Drugs

As of August 2013, both ATC and MeSH are integrated in RxNorm. We consider all MeSH drugs present in RxNorm, regardless as to whether they correspond to descriptors (also called “main headings”) or Supplementary Concept Records (SCR) in MeSH. Our starting set of ATC drugs consists of 5th-level ATC entities, from which we exclude combination drugs, often underspecified and unlikely to be represented in MeSH.

As a result of the integration of MeSH and ATC into RxNorm, the same RxNorm identifier is assigned to an ATC drug and to the equivalent drug in MeSH. Individual drugs in MeSH and ATC correspond to ingredients (IN) and precise Ingredients (PIN) in RxNorm. In order to facilitate the comparison of individual drugs between MeSH and ATC, we normalize the drugs by mapping each precise ingredient to its corresponding ingredient. We restrict our set of drugs to drugs of clinical relevance by filtering out those ingredients that are not associated with any clinical drugs in RxNorm. The set of individual drugs described here constitutes the set of eligible drugs for this study.

#### Drug classes

In order to minimize the number of pairwise comparisons between MeSH and ATC drug classes, we exclude broad, top-level classes from MeSH and ATC, for which the alignment would not be meaningful anyway. In practice, we exclude the 14 ATC classes of level 1 (anatomical classification). Similarly, we exclude the top-level descriptors of the *Chemicals and Drugs* hierarchy (i.e., D01-D27) in MeSH, as well as the top-level of the pharmacological action descriptors (*Pharmacologic Actions*, *Molecular Mechanisms of Pharmacological Action*, *Physiological Effects of Drugs*, and *Therapeutic Uses*). Additionally, we exclude 167 of the 1,241 ATC classes (2nd–4th level) corresponding to drug combinations, because combination drugs are often underspecified in ATC. We define drug combination classes in ATC as classes that contain “combination” (case-insensitive) in their labels or have ancestor classes with “combination” in their labels (e.g., *G03EA: Androgens and estrogens* are excluded along with their ancestor class *G03E: ANDROGENS AND FEMALE SEX HORMONES IN COMBINATION*). Finally, we further exclude from MeSH and ATC any classes that are not connected to any eligible individual drug (as defined above), directly or through a subclass (e.g., *A03AC: Synthetic antispasmodics, amides with tertiary amines* contains three drugs (*dimethylaminopropionylphenothiazine, nicofetamide, tiropramide)*, of which none are eligible^b^). The set of drug classes described here constitutes the set of eligible drug classes for this study.

#### Drug-class membership

**Figure 2:**
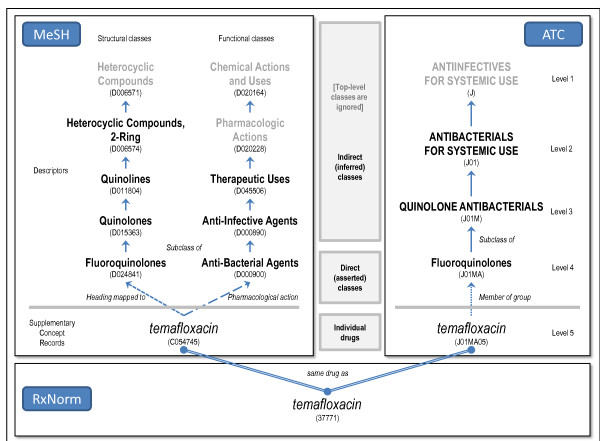
Individual drugs and drug classes in RxNorm, MeSH and ATC.

As mentioned earlier, the relation between a class and its drug members can be either direct (i.e., asserted) or indirect (i.e., inferred). In ATC, we consider as direct relations the relations asserted between 5th-level drugs and their 4th-level chemical classes. We infer drug-class relations between 5th-level drugs and the corresponding ATC classes at the 3rd and 2nd level. For example, as illustrated in Figure 2, the drug *temafloxacin* (J01MA05) is a member of the chemical class *Fluoroquinolones* (J01MA - asserted), the pharmacological class *QUINOLONE ANTIBACTERIALS* (J01M–inferred, 3rd level), and the therapeutic class *ANTIBACTERIALS FOR SYSTEMIC USE* (J01–inferred, 2nd level). Level-1 classes are ignored.

Extracting drug-class membership relations from MeSH is a more complex process, because drugs can be represented at different levels (descriptor or supplementary concept record), structural classes and functional classes are represented by different types of descriptors, and drugs are related to classes through various kinds of relationships. Relations between drugs (descriptors or SCRs) and functional classes (i.e., descriptor from the pharmacological actions hierarchy) are asserted through a “pharmacologic action” relationship. Relations between an SCR drug and its heading mapped to^c^ constitute the asserted relations to structural classes, as do relations between a descriptor drug and its direct parent. We infer drug-class relations between any drug and all the ancestors (direct or indirect) of the descriptors corresponding to their structural and functional (asserted) classes.

For example, as illustrated in Figure 2, the SCR *temafloxacin* has *Anti-Bacterial Agents* as pharmacological action and *Fluoroquinolones* as heading mapped to. From these asserted classes, we infer membership to *Anti-Infective Agents* (from *Anti-Bacterial Agents*) and to *Quinolones*, *Quinolines*, and *Heterocyclic Compounds, 2-Ring* (from *Fluoroquinolones*). Top-level classes are ignored.

### Aligning drug classes

#### Lexical alignment

We leverage the UMLS (synonyms and lexical matching features) for aligning drug classes by their names. In practice, we consider equivalent classes those MeSH and ATC classes, whose names map to the same UMLS concept. If both MeSH and ATC were integrated in the UMLS, we would only have to extract all UMLS concepts to which both a MeSH class and an ATC class are mapped. Since MeSH is integrated in the version of the UMLS used in this study, but ATC is not, we map ATC classes to the UMLS in order to link them to the equivalent classes in MeSH. More precisely, we use the *ExactString* and *NormalizedString* search function of the UTS API 2.0 to establish mappings between the names of the ATC classes and UMLS concepts. We use normalization only when the exact technique does not result in a match. We then associate the ATC class to a MeSH class through the UMLS concept to which they both map (e.g., *H03BA: Thiouracils* to *D013889: Thiouracil* through UMLS concept *C0039957*).

#### Instance-based alignment

We assess the similarity between two classes based on the individual drug members (instances) they share. In practice, we perform a pairwise comparison between all ATC classes and all MeSH classes, asserted and inferred. We define two scores for identifying equivalence and inclusion relations between ATC and MeSH classes.

##### Equivalence Score (ES)

The Jaccard coefficient (JC) is a measure of the similarity between two sets, for example between the set of drugs in a given ATC class (A) and in a given MeSH class (M). However, many drug classes only contain a small number of drugs, and, in this case, a small number of shared drugs between classes can yield relatively high Jaccard values. In order to reduce the similarity of pairs of classes with small numbers of shared drugs, we use a modified version of the Jaccard coefficient, JCmod, as suggested in [8],

$$\begin{array}{l} \mathrm{JC}\left(A,M\right)=\frac{\mathrm{am}}{a+m+\mathrm{am}}\\ \mathrm{ES}\left(A,M\right)=\mathrm{JCmod}(A,M)=\frac{\sqrt{\mathrm{am}\times \left(\mathrm{am}-0.8\right)}}{a+m+\mathrm{am}} \end{array}$$

where *am* represents the number of drugs common to A and M, and *a* + *m* + *am* the total number of unique drugs in both classes.

##### Inclusion Score (IS)

The Jaccard coefficient measures the similarity between the two classes, but does not reflect whether one class is included in the other. Because of the difference in organization and granularity between classes in ATC and MeSH, a given ATC class may not have an equivalent class in MeSH, but can be included in another MeSH class (e.g., *C07AA: Beta blocking agents, non-selective* included in *D000319: Adrenergic beta-Antagonists*). Such inclusion relations are crucial for a comprehensive alignment of the drug classes. We introduce a metric for finding fine-grained (“child”) classes that are included in coarse (“parent”) classes. This metric combines two elements. The first one measures the intensity of the “one-sidedness”, i.e., the extent to which the instances outside the intersection are not distributed between both sides, but rather belong to only one of the two classes. The second element measures the coverage of the finer-grained (“child”) class by the coarser (“parent”) class.

*IS* is calculated as follows:

$$\begin{array}{l} \mathrm{IS}\left(A,M\right)=0,\;\mathrm{for}\;C_{p\left(A,M\right)}=0\;\mathrm{and}\;C_{c\left(A,M\right)}=0\\ \mathrm{IS}\left(A,M\right)=\frac{a-m}{a+m}\times \frac{\mathrm{am}}{\min\left(\mathrm{am}+a,\mathrm{am}+m\right)},\;\mathrm{otherwise} \end{array}$$

where *am* represents the number of drugs common to A and M, and *a* and *m* the number of drugs specific to A and M, respectively.

For example, if A contains 10 drugs and M contains 20 drugs and if the two classes share 9 drugs, *IS(A,M)* = 0.75, providing a strong indication that A is included in M.

More generally, a value of *IS* close to 0 indicates that the drugs that are not shared by the two classes are evenly distributed between the ATC and MeSH class, i.e., there is no inclusion relation between the classes. In contrast, a value of *IS* close to 1 (in absolute value) indicates that the parent class contains most of the drugs that are not shared by the two classes and that the child class has a small proportion of specific drugs. The *IS(A,M)* score varies between -1 and 1, and a score of 1 corresponds to the inclusion of A in M, while a score of -1 corresponds to the inclusion of M in A.

##### Selecting classes with the best equivalence and inclusion relations

A given class in ATC or MeSH may have both equivalence and inclusion relations to classes from the other terminology. Moreover, it may have more than one equivalence relation and often has multiple inclusion relations. We propose an approach for selecting the best equivalence and inclusion relations for a given class. We heuristically determined 0.5 to be a reasonable threshold for both *ES* and *IS*. Therefore, none of the pairs of classes with *ES* or *IS* values lower than 0.5 will be considered for equivalence or inclusion, respectively. For a given class C_c_, the class C_e_ selected as the best equivalent class is the one with the highest *ES*. In contrast, the class C_p_ selected as the best inclusion class is not necessarily the one with the highest *IS*, because the class with the highest *IS* is most likely a very broad class. *IS* favors large parent classes, while the best parent class is the smallest parent class that covers a large proportion of the child class. Therefore, we select as the best inclusion relation the first pair among the best candidate equivalence pairs for which *IS* is above the threshold of 0.5. Although it might seem counterintuitive to select inclusion pairs among the candidate equivalence pairs, the high *ES* is consistent with the requirement for coverage of the child class by the parent class.

Usually the best equivalence and inclusion pairs are different, but not always. For instance, the mapping between two very similar classes, where one class contains a few specific drugs, might have both *IS* and *ES* above the threshold. Different use cases may call for different strategies for determining the best equivalent and inclusion pairs. For instance, while our strategy considers both scores, *ES* and *IS*, when they are above the threshold, an alternative strategy could be to choose one score over the other based on *max(ES, IS)*.

### Assessing the consistency between lexical and instance-based alignments

We hypothesize that classes with similar drugs should have similar names and classes with similar names should contain similar drugs. We compare the results of the lexical and instance-based alignment methods and assess their consistency. We expect the lexical alignment to identify equivalence classes, not class inclusion. Therefore, pairs of classes identified through the lexical alignment (LEX+) and identified as equivalent through the instance-based alignment (EQ+) are considered consistent, as are the pairs of classes neither identified through the lexical alignment (LEX-) nor identified as equivalent through the instance-based alignment (EQ-). Conversely, pairs of classes identified through the lexical alignment (LEX+) but not identified as equivalent through the instance-based alignment (EQ-) are considered inconsistent, as are the pairs of classes not identified through the lexical alignment (LEX-) but identified as equivalent through the instance-based alignment (EQ+).

## Results

### Establishing a common reference for drugs, drug classes and drug-class members

#### Drugs

As shown in Table 1, we retrieved from RxNorm 2,239 *Ingredients* (IN) and *Precise Ingredients* (PIN) that are mapped to 2,730 unique drugs in ATC, and 5,274 that are mapped to 4,153 drugs in MeSH. After normalization to INs, we selected 2,215 INs for ATC and 4,112 for MeSH. Finally, after restricting the RxNorm INs to those that are clinically relevant, we selected 1,706 INs for ATC and 2,339 for MeSH. Of these, 1,685 drugs are present in both ATC and MeSH.

**Table 1:** Selection of the ATC and MeSH classes suitable for the instance-based alignment

	**ATC**	**MeSH**
Candidate drugs in terminology	2,730	4,153
Corresponding drug entities in RxNorm (IN, PIN)	2,239	5,274
Drug entities after normalization of PINs to INs	2,215	4,112
Restriction to clinically-significant ingredients	1,706	2,339
Restriction to clinically-significant ingredients present in both terminologies	1,685	1,685

#### Drug classes

From the 1,255 ATC classes (1st–4th level) we excluded 14 ATC classes at the 1st level (anatomical classification) and 167 classes corresponding to drug combinations, leaving 1,074 classes eligible for the lexical alignment. We further excluded 81 empty classes without any drug (ATC contains empty classes by design), and 159 classes containing only drugs that cannot be mapped to RxNorm. The final set of ATC classes eligible for the instance-based alignment, A*, contains 834 drug classes, of which 558 are considered asserted (4th level) and 276 inferred (2nd–3rd level).

In MeSH, we identified 1,516 descriptors as drug classes for the eligible drugs, including 1,223 asserted classes and 293 inferred classes. These classes constitute the set of MeSH classes eligible for both the lexical and the instance-based alignment, M*. We classify 403 of the drug classes in M* as functional classes, i.e., their descriptors are located in the *Chemical Actions and Uses [D27]* sub-tree in MeSH, and 1,113 as structural classes.

#### Drug-class membership

For the 1,685 eligible drugs in MeSH, we established 15,122 drug-class pairs, of which 4,759 are asserted and 10,363 inferred. For the eligible drugs in ATC, we established 6,368 drug-class pairs, of which 2,140 are asserted and 4,228 inferred.

### Aligning drug classes

#### Lexical alignment

For the 1,074 eligible ATC classes, we were able to retrieve 226 mappings to descriptors from the *Chemicals and Drugs* ([D]) tree in MeSH. We found 18 mappings for therapeutic classes (2nd level), 43 for pharmacological classes (3rd level), and 165 for chemical classes (4th level). Of the 226 mappings, 99 are to pharmacological actions (functional classes) in MeSH, whereas 127 are to other descriptors at various levels of the MeSH hierarchy (structural classes).

#### Instance-based alignment

##### Equivalence and inclusion scores

Of the 834 ATC classes eligible for instance-based alignment (|A*| = 834), 828 (99%) could be associated with at least one MeSH class. Of the 1,516 eligible drug classes in MeSH (|M*| = 1,516), 1,317 (87%) could be associated with at least one ATC class. We conducted a pairwise comparison of all ATC classes with all MeSH classes (|A*| x |M*| = 1,264,344). For the 26,842 pairs that had at least one drug in common, we calculated the equivalence (*ES*) and inclusion (*IS*) scores. As shown in Table 2, 223 pairs (<1%) had an *ES* ≥ .5 and were considered equivalent (*EQ+*), and 6,257 pairs (23%) had an *IS* ≥ .5 and were considered in inclusion relation (*IN+*). Of note, there were 108 pairs with both strong equivalence and inclusion relations (*EQ +* and *IN+*). The remaining 20,470 pairs were considered unrelated, absent any strong equivalence or inclusion relations (*EQ- and IN-*).

**Table 2:** Analysis of the instance-based alignment between ATC and MeSH classes–equivalence vs. inclusion relations

		**Inclusion relation**		
		**Yes (IN+)**	**No (IN-)**	**Total**
**Equivalence relation**	Yes (EQ+)	108	115	**223**
	No (EQ-)	6,149	20,470	**26,619**
	**Total**	**6,257**	**20,585**	**26,842**

##### Classes with strong equivalence and inclusion relations

A given class in ATC or MeSH may have more than one strong relation to a drug class from the other terminology. We determined the best equivalence and inclusion mappings (not mutually exclusive) for each of the 828 ATC and 1,317 MeSH classes with shared drugs, respectively.

As shown in Table 3 (top), 828 ATC classes had some relation (equivalence or inclusion, but not necessarily strong) to a MeSH class. Of these, we identified 149 ATC classes (18%) with at a strong equivalence relation to MeSH, all but one of which also showed a strong inclusion to some MeSH class (albeit not necessarily the same as the equivalent class). A strong inclusion relation to MeSH was found for 728 (87%) of these ATC classes. On the other hand, 1,317 MeSH classes had some relation to an ATC class. Of these, we identified 165 MeSH classes (12%) with a strong equivalence relation to ATC, most of which also showed a strong inclusion relation to some ATC class. A strong inclusion relation to ATC was found for 510 (39%) of these MeSH classes (Table 3, bottom). The 1,317 MeSH classes linked to ATC include 374 functional classes (28%) and 943 structural classes (72%). Overall, a strong relation (equivalence or inclusion) was found between 729 ATC classes in ATC and the 555 MeSH classes.

**Table 3:** Characterization of the associations between ATC and MeSH classes based on scores for equivalence and inclusion

**ATC to MeSH**		**Best equivalence**		
		**>.5**	**<.5**	**Total**
**Best inclusion**	**>.5**	148 (17%)	580 (70%)	728 (87%)
	**<.5**	1 (1%)	99 (12%)	100 (13%)
	**Total**	149 (18%)	679 (82%)	828 (100%)
**MeSH to ATC**		**Best equivalence**		
		**>.5**	**<.5**	**Total**
**Best inclusion**	**>.5**	120 (9%)	390 (30%)	510 (39%)
	**<.5**	45 (3%)	762 (58%)	807 (61%)
	**Total**	165 (12%)	1,152 (88%)	1,317 (100%)

### Assessing the consistency between lexical and instance-based alignments

The results of the comparison between the lexical and instance-based alignments are shown in Table 4. We performed the comparison on the cross-product of the 834 eligible ATC and 1,516 MeSH classes (1,264,344 pairs). Of the 226 pairs of equivalent classes between ATC and MeSH identified through the lexical alignment, 36 (16%) were confirmed through the instance-based approach (LEX+/EQ+), of which 14 were also categorized as inclusion relations. Not surprisingly, no equivalence relation was identified by either approach for the bulk of the pairs from the cross-product between ATC and MeSH classes. A total of 313 inconsistencies between the two alignment approaches were identified, including 126 pairs identified exclusively by the lexical alignment (LEX+/EQ-), and 187 pairs specific to the instance-based alignment (LEX-/EQ+). This finding disproves our initial hypothesis that classes with similar names have similar drugs and vice versa. Of note, 64 pairs of equivalent classes identified through the lexical alignment were not amenable for processing by the instance-based alignment, because at least one class of the pair did not contain any eligible drug.

**Table 4:** Consistency between lexical and instance-based alignments of drug classes (italics values denote inconsistencies)

		**Lexical alignment**		
		**Yes (LEX+)**	**No (LEX-)**	**Total**
**Instance-based alignment**	**Yes (EQ+)**	36	*187*	223
	**No (EQ-)**	*126*	1,263,995	1,264,121
	**Total**	**162**	**1,264,182**	**1,264,344**
	**No data**	64		
	**Total LEX+**	**226**		

## Discussion

### Analysis of similarities and discrepancies between lexical and instance-based alignments

As illustrated through a few examples throughout this section, our framework facilitates the comparison of drug classes across sources and reveals inconsistencies in the classes, as well as deficiencies in the alignment techniques.

### Valid mappings

We identified an equivalence relation between the 4th-level ATC class *Tetracyclines* (J01AA) and the MeSH descriptor *Tetracyclines* (D013754). The two classes share nine drugs. The MeSH class has one extra drug (*meclocycline*), which is in a different class in ATC (*Antiinfectives for treatment of acne*), because, although structurally similar, it is not used systemically but topically. Jaccard similarity is high (0.86). This (equivalence) mapping is also identified by the lexical technique (exact match). Of note, the inclusion score (1 in absolute value) is also high, because there is only one drug that is not in common, which is - automatically - located on only one side of the intersection.

### Erroneous lexical mappings

We identified an inclusion mapping between the 4th-level ATC class *Fluoroquinolones* (S01AE) and the MeSH descriptor *Fluoroquinolones* (D024841). Although the two class names are identical, which would suggest an equivalence relation, our mapping is identified as an inclusion, with seven drugs in common, one drug specific to the ATC class and eleven drugs specific to the MeSH class. In fact, the ATC class is the specific class of fluoroquinolones for ophthalmic use (S01AE), in contrast to the class of fluoroquinolones for systemic use (J01MA)^d^. The fluoroquinolones used for eye disorders are (almost) a subset of all fluoroquinolones and the ATC class S01AE is appropriately characterized as being included in the MeSH class for fluoroquinolones. This example also constitutes an erroneous lexical mapping, since lexical mappings are expected to reflect equivalence relations.

### Missing instance-based mappings

Many ATC and MeSH classes share only one or very few drugs, making it difficult to assess equivalence or inclusion with confidence. For example, the 4th-level ATC class *Silver compounds* (D08AL) and the MeSH descriptor *Silver Compounds* (D018030) share only one drug (*silver nitrate*), where *Silver Compounds* (D018030) contains another drug (*silver acetate*), which is in RxNorm but not in ATC. The modified version of the Jaccard coefficient has a score of 0.22 in this case, which is below our threshold of 0.5 for equivalence. However, we classified the ATC class D08AL as being included in the MeSH class *Silver Compounds*.

During this failure analysis, we discovered that some MeSH drugs did not have a pharmacological action assigned to them as we expected. For example, while *pyrantel* is listed as *Antinematodal Agents*, *oxantel* is not^e^. The MeSH editorial rules require that a certain number of articles assert a given pharmacologic action for it to be recorded in MeSH. Because of these missing pharmacologic actions, the 3rd-level ATC class *ANTINEMATODAL AGENTS* (P02C) fails to be mapped to the MeSH pharmacological action *Antinematodal Agents* (D000969), the Jaccard similarity being below the threshold (0.37).

As mentioned earlier, some ATC classes only contain drugs that cannot be mapped to MeSH through RxNorm, which we used to bridge between the two. Such classes may be amenable to lexical alignment, but cannot be aligned through their instances. Similarly, some drug entities and biologicals (e.g., vaccines) are less well standardized than most common drugs. For this reason, the instance-based alignment may not be able to align these classes, when simple lexical techniques can. For example, the instance-based method fails to align the two classes *Epoxides* (L01AG) and *Epoxy Compounds* (D004852) because the ATC class does not contain any eligible drug (the only instance, *etoglucid* (L01AG01), is not listed as a clinical drug in RxNorm).

### Missing lexical mappings

Despite the use of UMLS synonymy and normalization, the lexical alignment fails to identify a mapping between the 3rd-level ATC class *POTASSIUM-SPARING AGENTS* (C03D) and the MeSH pharmacological action *Diuretics, Potassium Sparing* (D062865). In contrast, the instance-based alignment identifies an equivalence mapping with high Jaccard similarity (0.72). This finding is consistent with the conclusions of [7].

### Further characterization of equivalence and inclusion relations

Even when considering only strong relations and the best inclusion relations between ATC and MeSH classes, it is difficult to give a detailed account of the directionality of the relations, and the distribution between structural and functional classes. Some salient findings are summarized in Table 5. For example, we found 223 (strong) equivalence relations between 149 unique ATC classes and 165 unique MeSH classes, distributed almost evenly between structural and functional classes in MeSH. When restricting the analysis to the best inclusion relations, more ATC classes (728) are found to be included in some MeSH class, than MeSH classes (510) are in some ATC classes. And fewer functional classes (146) than structural classes (364) in MeSH are included in some ATC class.

**Table 5:** Detailed analysis of the mapping between ATC and MeSH classes–Structural vs. functional classes

**Type of relation**	**Direction**	**# strong relations**	**# unique ATC classes**	**# unique ATC classes**
**Equivalence (all)**	**ATC-MeSH (all)**	223	149	165
	**ATC-MeSH (St)**	115	77	84
	**ATC-MeSH (Fn)**	108	86	81
**Inclusion (all)**	**ATC to MeSH (all)**	4914	728	650
	**MeSH (all) to ATC**	1343	358	510
**Inclusion (best)**	**ATC to MeSH (all)**	1267	728	483
	**ATC to MeSH (St)**	597	559	275
	**ATC to MeSH (Fn)**	670	657	208
	**MeSH (all) to ATC**	568	264	510
	**MeSH (St) to ATC**	406	211	364
	**MeSH (Fn) to ATC**	162	102	146

Details of the instance-based alignment between functional (Fn) and structural (St) classes in ATC and MeSH.

For almost all drug classes in ATC that have an equivalence mapping to a drug class in MeSH, there is also at least one inclusion mapping to a broader class in MeSH. There is only one exception. The class *Drugs used in diabetics* (A10) is equivalent to *Hypoglycemic Agents* (D007004), which is already at the highest level we consider in MeSH (we ignore its parent class *Physiological Effects of Drugs* because it is too general). In contrast, there are 45 classes in MeSH that are equivalent to ATC classes but are not included in another class in ATC. For example, *Antiparkinson Agents* (D00978) maps to the 2nd level class *Anti-Parkinson Drugs* (N04) in ATC. Because we exclude 1st level classes in ATC, there is no parent class in ATC which would include the drug of the MeSH class *Antiparkinson Agents*. Conversely, the ATC class *Anti-Parkinson Drugs* (N04) is included in the higher level class *Central Nervous System Agents* (D002491) in MeSH, which is a parent of *Antiparkinson Agents*.

The alignment between ATC classes and MeSH classes can be further characterized, especially in order to account for concomitant occurrences of a strong inclusion relation to a structural class and to a functional class. As shown in Table 6, of the 505 strong equivalence and best inclusion relations to structural and functional classes in MeSH, the most frequent situation is the concomitant occurrence of inclusion to both a structural and a functional class. Of note, there is only one case where an equivalence relation occurs without a concomitant inclusion relation.

**Table 6:** Detailed analysis of the mapping between ATC and MeSH classes–equivalence vs. inclusion relations

**ATC to MeSH**	**To a structural class only**	**To a functional class only**	**To both a structural and a functional class**	**Total**
Equivalence relation only	0	1	0	**1**
Both equivalence and best inclusion relations	1	8	58	**67**
Best inclusion relations only	50	75	312	**437**
**Total**	**51**	**84**	**370**	**505**

Analysis of concomitant equivalence and best inclusion relations between ATC and MeSH classes, when structural and functional classes in MeSH are considered separately.

### Application of the framework to the alignment of important drug classes

One typical use case for the alignment of drug classes is to find equivalent classes in reference sources for a given class (e.g., to find which class best represents *macrolides* in MeSH and ATC). In order to illustrate how our approach supports the alignment of drug classes between MeSH and ATC, we applied our framework to a set of clinically relevant drug classes. We used the set of high-severity, clinically significant drug–drug interactions created by [17], in which most drugs are categorized in reference to drug classes.

We extracted all 13 drug classes from the list of verified critical drug–drug interactions discussed in their paper (Table 7). We first performed a lexical mapping to identify these 13 classes in MeSH and ATC (using normalized string matches against the UMLS). Only in six cases did the lexical mapping approach retrieve classes in both classifications. In another six cases, we were able to retrieve the class in either ATC or MeSH. The class *QT prolonging agents* was not found in either source.

**Table 7:** Lexical mapping to ATC and MeSH for 13 clinically relevant drug classes

**DDI class**	**ATC class lexical match**	**MeSH class lexical match**	**Best corresponding class in ATC**	**Best corresponding class in MeSH**
**Triptans**	-	*Tryptamines (D014363)*	*Selective serotonin (5HT1) agonists (N02CC)*	-
**Proton pump inhibitors**	*Proton pump inhibitors (A02BC)*	Proton pump inhibitors (D054328)	-	*2-Pyridinylmethylsulfinyl-benzimidazoles (D053799)*
**HMG CoA reductase inhibitors**	*HMG CoA reductase inhibitors (C10AA)*	*Hydroxymethylglutaryl-CoA Reductase Inhibitors (D019161)*	-	-
**Tricyclic antidepressants**	-	*Antidepressive agents, Tricyclic (D000929)*	*Non-selective monoamine reuptake inhibitors (N06AA)*	-
**Protease inhibitors**	*Protease inhibitors (J05AE)*	Protease inhibitors (D011480)	-	*HIV Protease inhibitors (D017320)*
**Narcotic analgesics**	-	*Narcotics (D009294)*	*OPIOIDS (N02A)*	-
**Selective serotonin reuptake inhibitors (SSRIs)**	Selective serotonin reuptake inhibitors (N06AB)	Serotonin uptake inhibitors (D017367)	*Selective serotonin reuptake inhibitors (N06AB)*	*Serotonin uptake inhibitors (D017367)*
**MAO inhibitors**	MAO inhibitors (C02KC)	*Monoamine oxidase inhibitors (D008996)*	*Monoamine oxidase inhibitors, non-selective (N06AF)*	Benzylamines (D001596)
**Macrolides**	Macrolides (J01FA)	Macrolides (D018942)	*Macrolides (J01FA)*	*Macrolides (D018942)*
**Azoles**	-	*Azoles (D001393)*	*Imidazole and triazole derivatives (D01AC)*	-
**Amphetamine derivatives**	-	Amphetamines (D000662)	-	-
**Ergot alkaloids and derivatives**	Ergot alkaloids (C04AE, G02AB, N02CA)	Ergot Alkaloids (D004876)	-	Ergotamines (D004879)
**QT prolonging agents**	-	-	-	-

Lexical mapping to ATC and MeSH (columns 2-3) for 13 clinically relevant drug classes, along with their corresponding class in the other source obtained through instance-based mapping (columns 4-5). Italicized classes denote best corresponding pairs of classes.

For each drug class that we retrieved through lexical mapping, we used our instance-based approach to determine the best corresponding class in the other terminology. Table 8 shows the strength of the mappings in terms of equivalence and inclusion. There is only one case (*HMG CoA reductase inhibitors*) where the two lexical matches also correspond to the best equivalent classes based on the drug instances. For five other classes we found equivalent class pairs starting from one lexical match. For four classes we could not find equivalent mappings across the two classifications, but inclusion mappings instead. Finally, three classes were left unmapped. (Two of these classes were underspecified as evidenced by the mention “[and] derivatives” in their name. The last one, *QT prolonging agents*, was not represented in either source, which is often the case for drug classes defined in reference to adverse effects [18]).

**Table 8:** Best corresponding classes in ATC and MeSH for 13 clinically relevant drug classes

**DDI class**	**ATC class**	**MeSH class**	**Drugs common**	**Drugs only in ATC**	**Drugs only in MeSH**	**ES.**	**IS**	**Rel.**
**Triptans**	Selective serotonin (5HT1) agonists	Tryptamines	7	0	1	0.82	-1	Eq
**Proton pump inhibitors**	Proton pump inhibitors	2-Pyridinylmethylsulfinyl-benzimidazoles	5	1	0	0.76	1	Eq
**HMG CoA reductase inhibitors**	HMG CoA reductase inhibitors	Hydroxymethylglutaryl-CoA Reductase Inhibitors	8	0	2	0.76	-1	Eq
**Tricyclic antidepressants**	Non-selective monoamine reuptake inhibitors	Antidepressive agents, Tricyclic	10	2	2	0.69	0	Eq
**Protease inhibitors**	Protease inhibitors	HIV Protease inhibitors	8	3	1	0.63	0.44	Eq
**Narcotic analgesics**	OPIOIDS	Narcotics	15	3	11	0.50	-0.48	Eq
**Selective serotonin reuptake inhibitors (SSRIs)**	Selective serotonin reuptake inhibitors	Serotonin uptake inhibitors	6	0	8	0.40	-1	In
**MAO inhibitors**	Monoamine oxidase inhibitors, non-selective	Monoamine oxidase inhibitors	3	0	5	0.32	-1	In
**Macrolides**	Macrolides	Macrolides	8	0	21	0.26	-1	In
**Azoles**	Imidazole and triazole derivatives	Azoles	11	1	147	0.07	-0.90	In
**Amphetamine derivatives**	-	-						-
**Ergot alkaloids and derivatives**	-	-						-
**QT prolonging agents**	-	-						-

Best corresponding classes in ATC and MeSH for 13 clinically relevant drug classes, with the equivalence (ES) and inclusion (IS) scores from our framework’s metrics, and the relation, equivalence or inclusion, between the two classes (Rel).

This application illustrates the effectiveness of our framework to support a clinical expert in the curation of an alignment of drug classes between MeSH and ATC. It helps identify lexically similar classes in these two sources, but, more importantly, it helps identify which class of the other source is most closely related to a given class. This feature enables experts to verify if the equivalence suggested through lexical mapping is also supported by a large proportion of shared drugs between these two classes. For example, the original class *Proton pump inhibitors* is mapped lexically to *Proton pump inhibitors* in ATC and to *Proton Pump Inhibitors* in MeSH. The best corresponding class in MeSH for the ATC class *Proton pump inhibitors*, however, is not *Proton Pump Inhibitors*, but rather *2-Pyridinylmethylsulfinyl-benzimidazoles*^f^. Moreover, in many cases, the original class can only be mapped lexically to either MeSH or ATC. In these cases, the instance-based mapping offers a solution for finding which class of the other source has the best correspondence. For example, the original class *Tricyclic antidepressants* can only be mapped lexically to the class *Antidepressive Agents, Tricyclic* in MeSH. However, the instance-based mapping identifies the ATC class *Non-selective monoamine reuptake inhibitors* as a potential equivalence.

While exploring mappings for these 13 clinically significant drug classes, we actually found no cases where the best corresponding classes in MeSH and ATC had exactly the same members. Here are some reasons why.

• As mentioned earlier, the classificatory principles used by ATC and MeSH are different. For example, *Azoles* represents a broad structural class in MeSH, whereas ATC splits azole drugs into several classes based on their therapeutic use (e.g., *antibacterials* and *antimycotics*).

• Some drugs appear to be missing from ATC, because of differences in the scopes of MeSH and ATC. Such drugs include dietary supplements (e.g., *red yeast rice*), veterinary drugs (e.g., many macrolides exclusively marketed for veterinary use), drugs of abuse (e.g., *heroin*) and drugs that only exist in combinations (e.g., *lopinavir and ritonavir*, but not *lopinavir* alone).

• Even though they are present in MeSH, some drugs appear to be missing from MeSH classes, because of missing relations to a drug class. For example, the class assigned to *tipranavir* is *Anti-HIV Agents*, while most of the drugs from the same ATC class are (more appropriately) in the MeSH class *HIV Protease Inhibitors*.

• In many cases, the name of an ATC class is underspecified, i.e., derives part of its meaning from its position in the hierarchy. As a consequence, the lexical mapping of such class names is likely to point to a broader class in MeSH. For example, the ATC class *Protease inhibitors* is under the class *Antivirals for systemic use*, which means that it represents not all protease inhibitors, but only those that are used to treat viral infections (which, in practice, means HIV infections.)^g^ In contrast, the MeSH class *Protease Inhibitors* truly represent all drugs, whose mechanism of action is to block some protease enzyme. Therefore, despite the similarity of their names, the ATC class *Protease inhibitors* is actually included in the MeSH class with the same name, and the best equivalence in MeSH for the ATC class *Protease inhibitors* is actually the class *HIV Protease Inhibitors*.

• Differences in granularity between MeSH and ATC classes are also responsible for some of the discrepancies observed in the mapping between the two sources. For example, the MeSH class *Monoamine Oxidase Inhibitors* is not found in ATC, which provides three more specific classes instead (*Monoamine oxidase inhibitors, non-selective*, *Monoamine oxidase A inhibitors*, *Monoamine oxidase B inhibitors*).

### Application of the framework to the integration of the MeSH and ATC classifications

**Figure 3:**
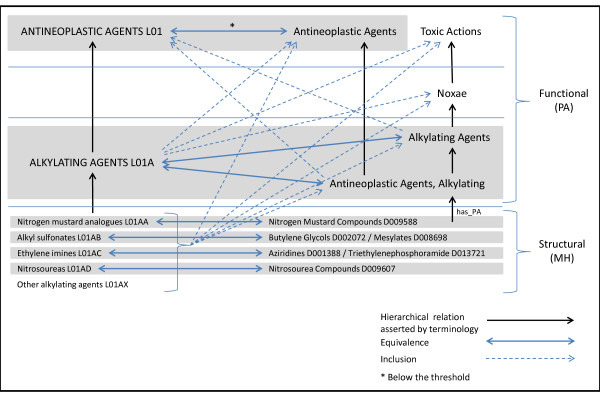
Integration of MeSH and ATC through the equivalence and inclusion relations obtained through our framework.

The equivalence and inclusion relations obtained through our framework can be combined in order to integrate the hierarchical structures of two drug classifications, such as MeSH and ATC. These additional relations create bridges across the original classifications, yielding an emerging hierarchy that combines both of them. As an illustration, we integrated the classes related to alkylating agents in MeSH and ATC. As depicted in Figure 3, all 4th-level classes under *Alkylating Agents (L01A)* in ATC have inclusion mappings to *Antineoplastic Agents, Alkylating* and *Alkylating Agents* in MeSH. The 3rd-level ATC class *Alkylating Agents (L01A)* itself is found to be equivalent to these two classes in MeSH and is included in their parent classes, *Antineoplastic Agents* and *Toxic Actions*, respectively. The 2nd-level ATC class *Antineoplastic Agents (L01)* can be regarded as equivalent to one of these parents, namely *Antineoplastic Agents*, although the equivalence score *ES* is slightly under the threshold of 0.5. Such a representation helps users make sense of the similarities and differences in the organizational structure of the classifications.

### Limitations and future work

The purpose of this framework is to provide a set of methods for assessing the consistency of drug classes across sources. While we believe our framework will facilitate the curation of an alignment of drug classes between two sources, it is beyond the scope of this work to provide such a reference alignment. Moreover, different reference alignments will most likely be required for different use cases, as different applications require different degrees of confidence.

As part of this framework, we have developed equivalence and inclusion scores, for which we have determined thresholds heuristically. We have not, however, fully investigated the impact of increasing or lowering these thresholds on the quality of the alignment. We plan to do so in future work.

Another limitation is that we have only applied our framework to one pair of drug classifications, MeSH and ATC. However, our framework is amenable to aligning any pairs of classifications for which instance-level data are available. We plan to revisit our earlier work on NDF-RT and SNOMED CT classes to demonstrate the generalizability of our approach.

As mentioned earlier, the instance-based alignment can be applied only to those classes for which both MeSH and ATC have drug members. This has been shown to be a limitation. On the other hand, the lexical alignment can still be used on these classes.

The UMLS Methesaurus relies for a large part on lexical similarity for determining synonymy among terms. With the recent inclusion of ATC in the UMLS Metathesaurus (in version 2013AB of the UMLS), it would no longer be necessary for us to perform the lexical alignment of ATC classes to MeSH classes, since we could simply derive it from the UMLS, where synonymous terms from various sources are given the same UMLS concept unique identifier. However, as discussed earlier, the lexical similarity of class names does not always reflect equivalence and our instance-based mapping remains an important alternative method for comparing classes.

## Conclusions

To our knowledge, our work is the first attempt to align drug classes with sophisticated instance-based techniques, while also distinguishing between equivalence and inclusion relations. Additionally, it is the first application of aligning drug classes in ATC and MeSH. Moreover, this is the first systematic investigation of the consistency between lexical and instance-based alignment techniques for these two drug resources. We believe that the proposed framework will effectively support the curation of a mapping between ATC and MeSH drug classes by providing a detailed account of the interrelations between the two resources.

## Endnotes

^a^ATC was integrated for the first time in version 2013AB of the UMLS released after this study was completed.

^b^None of these drugs are currently available on the U.S. market.

^c^If the SCR is mapped to a drug, rather than a structural class descriptor, we associate it with the structural class of this drug descriptor instead.

^d^When ATC was integrated into the UMLS Metathesaurus, new terms were created for ambiguous classes such as *Fluoroquinolones*, which appears at several locations in the ATC hierarchy with slightly different meanings (e.g., *Fluoroquinolone antiinfectives, ophthalmologic* for S01AE and *Fluoroquinolone antibacterials, systemic* for J01MA).

^e^The pharmacological action *Antinematodal Agents* for *oxantel* was not present in MeSH 2013, but was added to MeSH in the 2014 edition.

^f^Upon investigation, it appears that some proton pump inhibitor drugs, such as esomeprazole, were missing a link to the class *Proton Pump Inhibitors* in the 2013 version of MeSH. This was corrected in the 2014 version.

^g^When ATC was integrated into the UMLS Metathesaurus, the new term *Protease inhibitors, direct acting antivirals* was created for the underspecified class *Protease inhibitors* (J05AE).

## Competing interests

The authors declare that they have no competing interests.

## Authors’ contributions

RW and OB conceived the project and contributed equally to performing the acquisition, analysis, and interpretation of data and to the writing of the manuscript. Both authors read and approved the final manuscript.
